# The deletion of the EP402R and MGF505/360 genes attenuates a genotype I/II recombinant ASFV but fails to confer complete protection against homologous or genotype II challenge in pigs

**DOI:** 10.1080/22221751.2025.2608396

**Published:** 2026-01-12

**Authors:** Yao Li, Yingnan Liu, Zhuyun Sun, Zhenhua Xie, Chuanwen Tian, Rongrong Wang, Jun Gao, Maomao Wang, Jingyi Liu, Heng Wang, Guihong Zhang, Jie Li, Dongdong Di, Lang Gong, Hongjun Chen

**Affiliations:** a Chinese Academy of Agricultural Sciences, Shanghai Veterinary Research Institute, Shanghai, People’s Republic of China; b State Key Laboratory of Veterinary Public Health and Safety, College of Veterinary Medicine, China Agricultural University, Beijing, People’s Republic of China; c The Spirit Jinyu Biological Pharmaceutical Co. Ltd., Hohhot, People’s Republic of China; d Guangdong Provincial Key Laboratory of Zoonosis Prevention and Control, College of Veterinary Medicine, South China Agricultural University, Guangzhou, People’s Republic of China

**Keywords:** African swine fever virus, gene deletion, cross-protection, genotype I and II, EP402R

## Abstract

African swine fever (ASF), a pig disease caused by ASFV, is highly contagious and often lethal. The recent emergence of novel ASFV with I/II genomic recombination has posed significant challenges to global ASF prevention and control. In this study, we used the Chinese I/II genomic recombinant virulent strain ASFV-HN10005 (ASFV-HN) to construct two gene-deleted viruses. ASFV-HNΔMGF lacks the *MGF505-1R*–*MGF360-14L* genes cluster (including *MGF505-1R, -2R, -3R*, and *MGF360-12L, -13L, -14L*), and ASFV-HNΔCD2vΔMGF lacks both *EP402R* and the *MGF505-1R*–*MGF360-14L* genes cluster. ASFV-HNΔMGF still showed pathogenicity in domestic pigs, while ASFV-HNΔCD2vΔMGF showed markedly attenuated virulence, with all inoculated pigs surviving. However, these pigs did not gain complete protection against subsequent lethal challenge from the parental ASFV-HN strain or the virulent II-type strain ASFV-GZ. This implies that I/II genomic recombinant ASFV may have unique biological traits and immune evasion mechanisms. Our findings indicate the need to reassess current ASFV control strategies and provide a basis for future vaccine target selection.

## Introduction

ASF is a highly contagious and lethal hemorrhagic disease, characterized by a mortality rate approaching 100% in both domestic pigs and wild boars (www.woah.org). The disease is caused by the ASFV, which is the sole member of the genus Asfivirus within the family Asfarviridae and is classified as a large double-stranded DNA virus [1]. In China, genotype II ASFV was first detected in 2018, and by 2021, genotype I ASFV was also reported, resulting in the coexistence of both genotypes within the country [2,3]. Indeed, in 2022, recombinant strains of genotype I and II ASFV were first observed in China, and subsequently, similar recombinant strains were reported in Vietnam (2023) and Russia (2024) [4–6]. These recombinant strains maintain their virulence without any signs of attenuation, highlighting the urgent need to evaluate existing strategies to combat this emerging virus.

The emergence of recombinant ASFV presents significant challenges for prevention, diagnosis, and epidemiological tracking. To date, no effective commercial vaccine is widely available for the global prevention of ASF. Although live attenuated vaccines have been licensed in Vietnam, their broader application and efficacy await further evaluation. [7–11]. Over the past decades, various vaccine strategies have been explored, with particular focus on gene-deleted ASFV strains as potential attenuated live vaccines. Several genes implicated in ASFV pathogenesis, such as *E184L*, *A137R*, *I73R*, *EP402R* (CD2v), *L7L–L11L*, *MGF110*, *MGF505* (including *MGF505-1R*, *MGF505-2R*, *MGF505-3R*, and *MGF505-7R*), *MGF360* (comprising *MGF360-12L*, *MGF360-13L*, and *MGF360-14L*), *I177L*, and others, have been identified. Deleting these genes has shown promise in developing attenuated live vaccines by providing reliable protection against homologous virulent strains [8,11–16]. The construction of a multitarget attenuated live vaccine based on the *MGF505/360* genes cluster has been demonstrated to be feasible by several research teams [16–18]. Previous experiments have demonstrated that the HLJ/18 mutant strain, which lacks both the *EP402R* gene and six *MGF505/360* genes, confers strong immune protection against both the homologous virulent strain and genotype II variants [19,20]. Consequently, the *EP402R* and *MGF505/360* genes represent compelling candidate targets for multiple gene deletions.

In this study, we used the genotype I/II recombinant African swine fever virus strain ASFV-HN as the parental backbone to engineer targeted deletions in the *EP402R* gene and the *MGF505-1R*–*MGF360-14L* genes cluster (*MGF505-1R, -2R, -3R and MGF360-12L, -13L, -14L*). Two mutant viruses were successfully generated: ASFV-HNΔMGF, which lacks the *MGF505-1R*–*MGF360-14L* genes cluster, and ASFV-HNΔCD2vΔMGF, carrying deletions of both *EP402R* and the *MGF505-1R*–*MGF360-14L* genes cluster. ASFV-HN proved to be highly virulent, inducing acute ASF in domestic pigs. Deletion of the *MGF505-1R*–*MGF360-14L* genes cluster alone failed to markedly attenuate virulence. Substantial attenuation was observed only when *EP402R* and the *MGF360/505* genes cluster were simultaneously deleted. Importantly, pigs immunized with ASFV-HNΔCD2vΔMGF did not mount effective protection against subsequent challenge with the parental virulent strain or with the genotype II virulent strain ASFV-GZ.

## Materials and methods

### Biosafety statement

Animal experiments and ASFV infection were performed at the Animal Biosafety Level 3 (ABSL-3) facilities of Spirit Jinyu Biological Pharmaceutical Co., Ltd., in strict compliance with animal ethics guidelines. These experiments were approved by the relevant institutional ethics committee (Approval Nos: JY/ABSL3/IV/A/416/23011012, JY/ABSL3/IV/A/416/24010710, and JY/ABSL3/IV/A/416/25010705). Furthermore, the facility and experimental procedures are accredited by the China National Accreditation Service for Conformity Assessment (CNAS) (License No: CNAS-BL0101) and recognized by the Ministry of Agriculture and Rural Affairs.

### Virus and cells

As previously described [21], primary porcine bone marrow-derived macrophages (BMDMs) were prepared from 40-day-old piglets that were confirmed in the laboratory to be negative for common pig viruses, including porcine reproductive and respiratory syndrome virus (PRRSV), classical swine fever virus (CSFV), African swine fever virus (ASFV), and porcine parvovirus (PPV). These BMDMs were cultured in RPMI-1640 medium, which was supplemented with 10% fetal bovine serum (FBS), 1% Penicillin–Streptomycin (Pen Strep), 2 mM L-glutamine, and 10 ng/mL recombinant porcine GM-CSF (R & D Systems, Minneapolis, MN, USA), and maintained at 37℃ in a humidified atmosphere with 5% CO_2_. The highly virulent genotype II strain ASFV-GZ201801 (ASFV-GZ) and the genotype I/II recombinant virus ASFV-HN10005 (ASFV-HN) were obtained from the National African Swine Fever Regional Laboratory at South China Agricultural University. The ASFV-GZΔECM3 (an attenuated strain with deletions in *EP153R*, *CD2v*, *MGF360-12L*, *MGF360-13L*, and *MGF360-14L*) was maintained in our laboratory [22]. All viruses were propagated in BMDMs. Initial titration of the parental ASFV-HN virus showed no significant difference in viral titres between the HAD_50_ and TCID_50_ assays. Because the HAD_50_ assay is inapplicable to the non-hemadsorbing ASFV-HNΔCD2vΔMGF mutant, the TCID_50_ method was adopted for all subsequent titrations in primary BMDMs via the Reed-Muench method, ensuring a unified and consistent quantification approach across the study. [21,22].

### Homologous recombination

**Figure 1.**
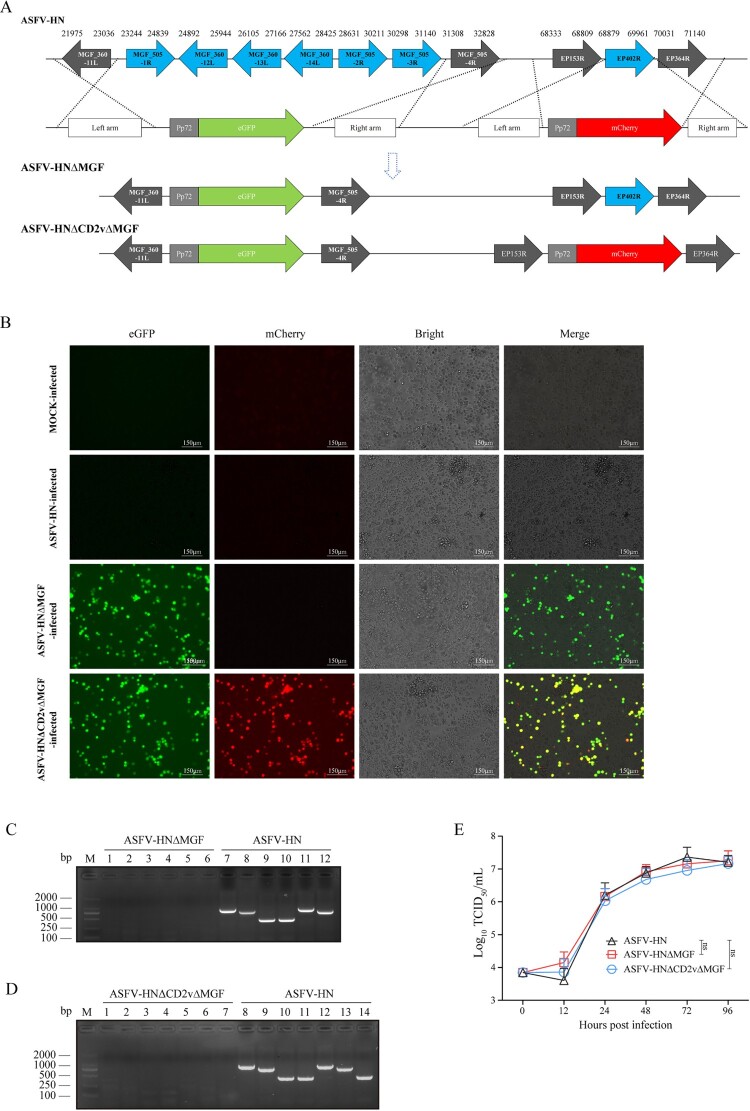
Illustrating the generation of various gene-deleted ASFV strains. (A) The deleted gene fragment was replaced with the *Pp72-eGFP* or *Pp72-mCherry* expression cassette. (B) Fluorescence from eGFP and mCherry confirmed that bone marrow-derived macrophages (BMDMs) were infected with ASFV-HNΔMGF (eGFP) or ASFV-HNΔCD2vΔMGF (eGFP and mCherry) viruses. Scale bar = 150 μm. (C) PCR confirmed the ASFV-HNΔMGF deletion mutant. Lane M shows the DNA molecular weight marker (DL2000), lanes 1–3 and 7–9 tested the *MGF360* genes cluster (*MGF360-12L*, *13L*, and *14L*), while lanes 4–6 and 10–12 tested the *MGF505* genes cluster (*MGF505-1R*, *2R*, and *3R*). (D) PCR confirmed the ASFV-HNΔCD2vΔMGF deletion mutant. Lane M shows the DNA molecular weight marker (DL2000), lanes 1–3 and 8–10 tested the *MGF360* genes cluster, lanes 4–6 and 11–13 tested the *MGF505* genes cluster, and lanes 7 and 14 tested the *EP402R* gene. (E) The in vitro growth characteristics of ASFV-HNΔMGF, ASFV-HNΔCD2vΔMGF, and the parental ASFV-HN viruses were evaluated using primary porcine macrophage cultures. These cultures were infected with each virus at a multiplicity of infection (MOI) of 0.1. Viral titres were subsequently measured at specified time points post-infection. The data are presented as means ± the standard deviations (SD). ns: no significant. The expected sizes of the PCR products are as follows: MGF360-12L(1153 bp), MGF360-13L(861 bp), MGF360-14L(472 bp), MGF505-1R(472 bp), MGF505-2R(1024 bp), MGF505-3R(934 bp), EP402R(473 bp).

The generation of gene-deleted ASFV was achieved through homologous recombination between the ASFV-HN genome and a recombinant transfer vector. The transfer vector, as illustrated in Figure 1, was constructed using fusion polymerase chain reaction (PCR). It includes flanking genomic regions (1.2 kb) of the target gene and a reporter gene cassette, which contains either mCherry or eGFP driven by the ASFV p72 late gene promoter *(Pp72*). This cassette was strategically inserted between the left and right arms to facilitate the selective removal of the target gene. BMDMs were transfected with 2 μg of the recombinant transfer vector using JetPEI® (Polyplus-transfection Inc., Illkirch, France). Subsequently, these cells were infected with ASFV-HN at a multiplicity of infection (MOI) of 0.1 at 6–8 h post-transfection. Fluorescent cells were identified using a fluorescence microscope and subsequently passage into fresh BMDMs. To verify the absence of the parental ASFV-HN and confirm the presence of the desired deletions in each recombinant genome, viral DNA was extracted from the virus stock and analyzed through PCR and sequencing. For viral whole-genome sequencing, the specific PCR and high-fidelity reaction systems were employed to amplify the ASFV genomic fragments via PCR. Subsequently, the Sanger DNA sequencing method, previously described in the literature, was utilized for sequencing [4].

### Animal trials

Commercial pigs ((Landrace, 10–15 kg)) were procured from a local farm that maintained the highest standards of biosafety and hygiene. These pigs were initially screened for common pig viruses through PCR or quantitative PCR (qPCR). To determine the 50% pig lethal dose (PLD_50_) of ASFV-HN, six groups of four pigs each were intramuscularly (i.m.) inoculated with 1 mL of the virus at 10-fold serial dilutions, ranging from 10^0^ to 10^5^ HAD_50_ infectious units. Following inoculation, mortality rates were monitored daily for 28 consecutive days. The PLD_50_ was then calculated based on the observed mortality data, using the method described by Reed and Muench[19]. To assess the virulence of ASFV-HN and gene-deleted ASFV-HN (ASFV-HNΔMGF and ASFV-HNΔCD2vΔMGF), the experimental animals were inoculated with either 10^3.0^ or 10^5.0^ TCID_50_ of the virus. Throughout a 28-day period, the rectal temperature (℃) and mortality rates (%) of pigs were systematically monitored, and clinical symptoms were meticulously documented [22,23]. Samples, including blood serum, heparin-treated-whole blood, oral swabs, nasal swabs, and rectal swabs, were collected at intervals of 0, 3, 5, 7, 9, 14, 21 and 28 days post-inoculated and analyzed for viral DNA via qPCR. Additionally, necropsies were conducted, during which the tonsils, liver, spleen, kidneys, heart, lungs, thymus, and lymph nodes were examined and scored for qPCR analysis as previously outlined [22,23].

To assess the protective efficacy of ASFV-HNΔCD2vΔMGF in domestic pigs (Landrace), 31 healthy pigs were included in this study (Figure 5(A)). Ten pigs (Group-I and Group-II) received a primary intramuscular immunization with 10^5.0^ TCID_50_ of ASFV-HNΔCD2vΔMGF, while another ten pigs (Group-III and Group-IV) were given phosphate-buffered saline (PBS) as controls. Additionally, five pigs were immunized with ASFV-GZΔECM3 (Group-V), a strain previously shown to provide effective homologous protection. All pigs received a booster immunization with the same dose 14 days after the primary immunization. This 14-day interval was chosen based on the immune response kinetics observed in our previous studies [23]. Six naïve pigs (Group-VI and Group-VII) were co-housed with the immunized groups (Group-I and Group-II) to serve as sentinels for monitoring viral shedding. On day 42 post-immunization (28 days after the booster), Groups-I and -III were intramuscularly challenged with 10^2.0^ TCID_50_ of the parental ASFV-HN strain, while Groups-II, -IV, and -V were challenged with 10^2.0^ TCID_50_ of the ASFV-GZ strain. All animals underwent necropsy 28 days post-challenge. Throughout the study, body temperature and clinical signs were monitored daily. Anticoagulated blood samples, along with oral, nasal, and anal swabs, were collected and analyzed using established methods [21].

### Quantitative PCR assay

Genomic DNA was extracted from various sample types, including tissue homogenates (Tissuelyser-FEII, Shanghai, China), oral swabs, nasal swabs, rectal swabs, and peripheral blood, using the GlinX Viral Nucleic Acid Extraction Kit (GlinX, Shanghai, China). Subsequently, quantitative PCR (qPCR) targeting the ASFV *p72* gene was conducted on the QuantStudio 5 system (Applied Biosystems, USA) following the manufacturer’s protocol [22].

#### Indirect enzyme-linked immunosorbent assay (ELISA)

In strict accordance with the manufacturer’s instructions, a cytokine enzyme-linked immunosorbent assay kit (Solarbio) was adopted to detect cytokines in porcine serum. The minimum detectable concentrations for IL-6 (SEKP-0004), TNF-α (SEKP-0009), IFN-α (SEKP-0045), IL-2 (SEKP-0002), and IFN-γ (SEKP-0010) were precisely 31.25, 4, 9, 62.5, and 4 pg/mL, respectively. The ASF blocking antibody detection kit (INGENNA, Madrid, Spain) was exploited to detect p72 antibodies, and the blocking percentage (S/N%) was computed. S/N% values of ≤ 40%, 40–50%, and ≥ 50% were respectively identified as negative, suspicious, and positive. The detection level of p30 and p54 antibodies referred to the methods previously delineated [15].

### Statistical analysis

GraphPad Prism (GraphPad Software Inc., La Jolla, USA) was utilized for all statistical analyses. The results are reported as means ± standard deviation (SD). Group comparisons were made using an unpaired, two-tailed Student’s *t*-test. For all tests, statistical significance was defined as a *p*-value < 0.05.

## Results

### Generation of ASFV-HNΔMGF and ASFV-HNΔCD2vΔMGF deletion mutants

After establishing the genomic background of the parental ASFV-HN strain – a genotype I/II recombinant closely resembling JS/LG/21 [4], as detailed in Figure S1(A), Table S1, and Table S2 – we further confirmed that the *EP402R* gene and the *MGF505-1R–MGF360-14L* gene cluster, targeted for deletion in this study, are entirely derived from genotype II strains (Figure S2(A) and Table S3). Using DNA homologous recombination technique we generated two different mutant viruses: ASFV-HNΔMGF, with deletions in the *MGF505-1R*–*MGF360-14L* genes cluster, and ASFV-HNΔCD2vΔMGF, with deletions in both the *EP402R* and *MGF505-1R*–*MGF360-14L* genes cluster, respectively (Figure 1(A)). The gene-deleted ASFV bearing one or two of the reporter genes *eGFP* and *mCherry* were purified through successive rounds of plaque purification and limiting dilution assays in primary BMDMs. Following successful infection, PCR analysis confirmed both the absence of the parental ASFV-HN virus and the presence of the intended deletions (Figure 1(B–D)). The precise genetic modifications and genomic stability of the resultant mutants were validated by whole-genome sequencing following clonal purification (Table S4 and Table S5). We compared the growth kinetics of the two gene-deleted ASFV to that of ASFV-HN via multistep growth curve analysis (Figure 1(E)). The growth patterns of the two gene-deleted ASFV were similar to those of the parental strain (ASFV-HN), with no significant differences observed. These findings suggest that the deletion of *MGF505-1R–MGF360-14L* genes cluster and/or the *EP402R* gene does not substantially impair the replicative capacity of ASFV-HN in porcine macrophages.

### Virulence evaluation of ASFV with different gene deletions

**Figure 2.**
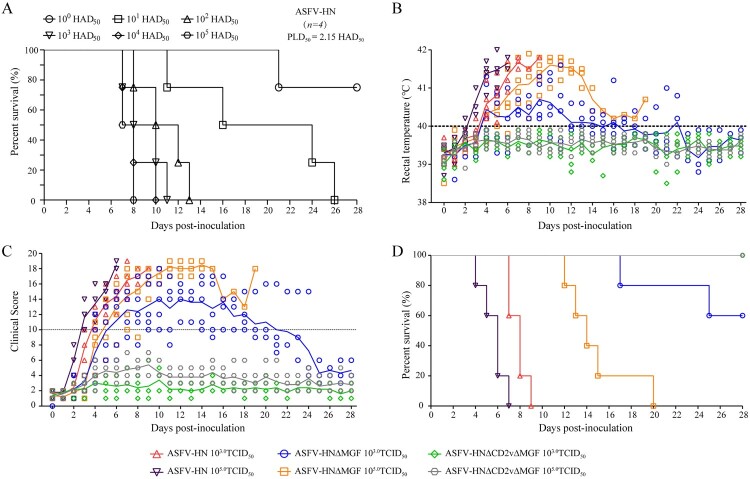
In vivo evaluation of different gene-deleted ASFV strains. (A) Survival rates of pigs inoculated with the wild - type ASFV-HN. (B) Rectal temperatures of pigs inoculated with ASFV-HNΔMGF, ASFV-HNΔCD2vΔMGF, and parental ASFV-HN strains. (C) Clinical scores recorded and calculated in pigs post-inoculation. (D) Survival rates of pigs following inoculation.

To determine whether these two gene-deleted ASFV were attenuated in pigs, we first evaluated the 50% pig lethal dose (PLD_50_) of the parental strain ASFV-HN in domestic pigs (10–15 kg). As shown in Figure 2(A), the PLD_50_ of ASFV-HN via intramuscular injection (IM) was 2.15 HAD_50_. Subsequently, a second animal trial was performed to evaluate the virulence of ASFV-HNΔMGF and ASFV-HNΔCD2vΔMGF in pigs. Domestic pigs (10–15 kg) were inoculated intramuscularly (IM) with 10^3.0^ and 10^5.0^ TCID_50_ of either the gene-deleted ASFV or parental ASFV-HN strain (*n* = 5 per group). Following infection, the body temperature and survival were monitored for 28 days. Additionally, the symptoms were scored using a previously described clinical scoring system [21,23]. Pigs inoculated with either ASFV-HNΔMGF or parental ASFV-HN developed an increased body temperature (>40℃) at 2–4 days post-inoculation (dpi), followed by the appearance of clinical signs associated with classical ASF, including ataxia, depression, anorexia, purple skin discoloration, and diarrhea (Figure 2 (B and C) and Table S6). Signs of the disease severity worsened progressively, and all pigs infected with ASFV-HN died within 10 dpi. The pigs infected with a high dose of (10^5.0^ TCID_50_) ASFV-HNΔMGF survived longer but still died within 20 days. Among those infected with a low dose of (10^3.0^ TCID_50_) ASFV-HNΔMGF, two pigs died at 17 and 25 dpi, respectively, while the remaining three survived until the end of the observation period (Figure 2(D)). Conversely, regardless of the dose, all pigs inoculated with ASFV-HNΔCD2vΔMGF remained healthy and survived for the whole immunization observation period (28 days) (Figure 2(B)–(D) and Table S6).

**Figure 3.**
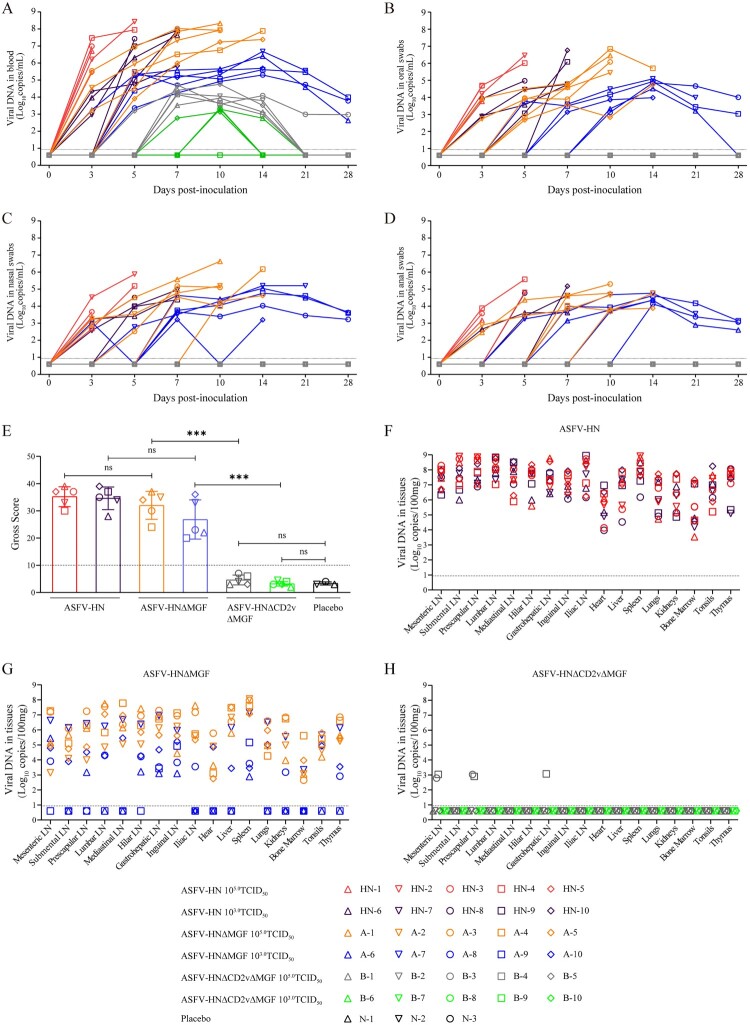
Dynamics of virus shedding and tissue pathology following infection with gene-deleted ASFV strains. (A) The viremia in pigs after inoculation. (B) The virus shedding in oral swabs from pigs after inoculation. (C) The virus shedding in nasal swabs from pigs after inoculation. (D) The virus shedding in anal swabs from pigs after inoculation. (E) Pathogenic scores for all pig groups were calculated and compared to the placebo control group. (F–H) Replication of ASFV-HN, ASFV-HNΔMGF, and ASFV-HNΔCD2vΔMGF in pigs. Lymph nodes (LN), thymus, bone marrow, and organ tissues were collected from deceased or euthanized pigs and subjected to viral DNA detection. The data are presented as means ± the standard deviations (SD). ***p *<* *0.001*, ns: no significant. Limit of detection (LOD) = 10^0.94^ copies/mL.

ASFV genomic DNA in the whole blood of the pigs was examined on 0, 3, 5, 7, 10, 14, 21, and 28 dpi. As shown in Figure 3(A), all pigs infected with either ASFV-HN or ASFV-HNΔMGF exhibited pronounced viremia by 3 dpi, with viral loads in the blood increasing sharply as the infection progressed, reaching maximal levels (10^5.45^–10^8.43^copies/mL) prior to death. Moreover, in the low-dose ASFV-HNΔMGF group, the three surviving pigs (A6, A8 and A9) exhibited peak viremia levels at 14 dpi, which subsequently declined gradually and fell below 10^3.99^ copies/mL by the end of the observation period (28 dpi). In contrast, the pigs in the ASFV-HNΔCD2vΔMGF group showed delayed viremia, occurring between 5 and 7 days, with lower viral DNA copies in the blood (10^2.76^–10^4.76^copies/mL) compared to those infected with ASFV-HN or ASFV-HNΔMGF (Figure 3(A)). Notably, at 21 and 28 dpi, one pig (B3) in the high-dose of ASFV-HNΔCD2vΔMGF group (10^5.0^ TCID_50_) exhibited lower levels of viremia, with viral loads 10^2.97^–10^2.99^ copies/mL. At the same time points, viral DNA was undetectable in the blood of the low-dose (10^3.0^ TCID_50_) inoculation group, indicating that the attenuated virus could be cleared within the 28-day observation period. Viral shedding was assessed at 0, 3, 5, 7, 10, 14, 21, and 28 dpi via oral, nasal, and anal swabs (Figure 3(B–D)). Viral DNA detection revealed that some pigs infected with ASFV-HN and ASFV-HNΔMGF exhibited viral shedding starting from 3 dpi, with levels reaching up to 10^3.17^–10^6.84^ copies/mL prior to death. In the ASFV-HNΔMGF-inoculated group, the three surviving pigs (A6, A8 and A9) showed a gradual decline in shedding levels from 14 to 28 days, with 10^2.60^–10^4.00^ copies/mL detected on day 28. In contrast, virus copy numbers in the oral, nasal, and anal swabs of pigs inoculated with ASFV-HNΔCD2vΔMGF remained below the detection limit (10^0.94^ copies/mL) at all time points (Figure 3(B–D)). Taken together, these results indicate that ASFV-HNΔMGF is slightly attenuated in pigs, and ASFV-HNΔCD2vΔMGF is dramatically attenuated in pigs.

**Figure 4.**
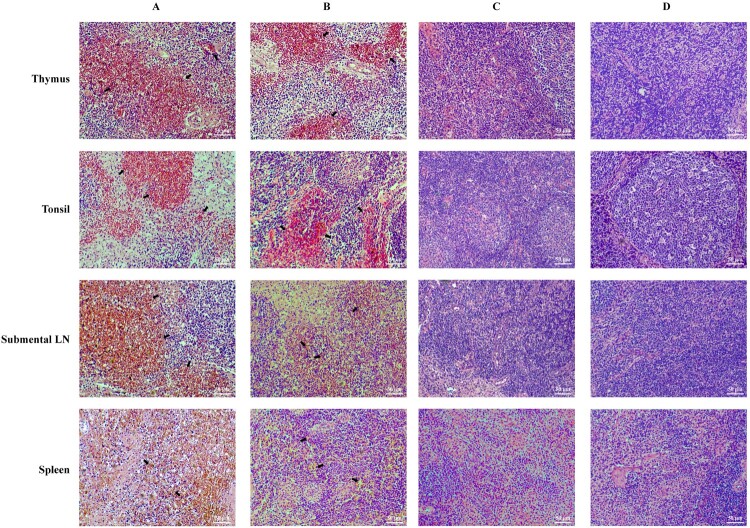
Histopathological sections (H&E staining) of the thymus, tonsil, submental lymph nodes, and spleen. The organs and tissues were collected from the pigs inoculated with 10^5.0^ TCID_50_ of ASFV-HN (Column A), ASFV-HNΔMGF (Column B), and ASFV-HNΔCD2vΔMGF (Column C). Column D, the tissues were collected from the pigs inoculated with placebo. LN: lymph nodes. Scale bar = 50 μm.

Necropsies were conducted on animals inoculated with the virus, either at the time of death or post-euthanasia. Gross pathological changes in organs and lymph nodes were assessed and scored using a previously established system, as detailed in Table S7 and Figure 3(E). This evaluation included tonsillar damage, lymph node congestion, and other pathological changes. All deceased pigs exhibited typical symptoms of acute ASF and corresponding gross lesions. Three pigs (A6, A8 and A9) survived inoculation with low-dose (10^3.0^ TCID_50_) of ASFV-HNΔMGF, but their spleens and lymph nodes showed varying levels of congestion and hemorrhage. High levels of viral DNA were detected in tissue samples from all animals infected with ASFV-HN and ASFV- HNΔMGF (Figure 3(F)–(H)). Conversely, the vast majority of pigs inoculated with ASFV-HNΔCD2vΔMGF exhibited no gross pathological changes. Two pigs (B3 and B4) inoculated with a high-dose (10^5.0^ TCID_50_) ASFV-HNΔCD2vΔMGF displayed varying degrees of lymph node enlargement, yet no gross lesions were observed in the parenchymal organs. In the low-dose group, only one pig (B7) showed mild swelling lymph nodes, which may reflect individual variation. Additionally, in the group inoculated with high-dose of ASFV-HNΔCD2vΔMGF, low levels of viral DNA were detected in the lymph nodes of two pigs (B3 and B4, 10^2.79^–10^3.07^ copies/mL) (Figure 3(H)). By the end of the observation period, no viral DNA was detected in any tissues of the surviving pigs that received a low-dose of ASFV-HNΔCD2vΔMGF. Histopathological examinations were conducted on major immunological organs and tissues, including the spleen, thymus, tonsils, and submental lymph nodes, with representative findings illustrated in Figure 4(A)–(D). Hemorrhagic lymphocyte necrosis (indicated by black arrows) was consistently observed in the spleen, thymus, tonsils, and submental lymph nodes of all deceased pigs (Figure 4 (A) and (B)). In contrast, pigs inoculated with the ASFV-HNΔCD2vΔMGF gene-deleted virus or inoculated with the placebo exhibited no significant lymphocyte hemorrhage or necrosis in these lymph node regions (Figure 4 (C) and (D)). These findings indicate that the replication level of ASFV-HNΔCD2vΔMGF in pig tissues is significantly reduced, thereby preventing severe tissue damage.

### Incomplete protection in ASFV-HNΔCD2vΔMGF-immunized pigs

**Figure 5.**
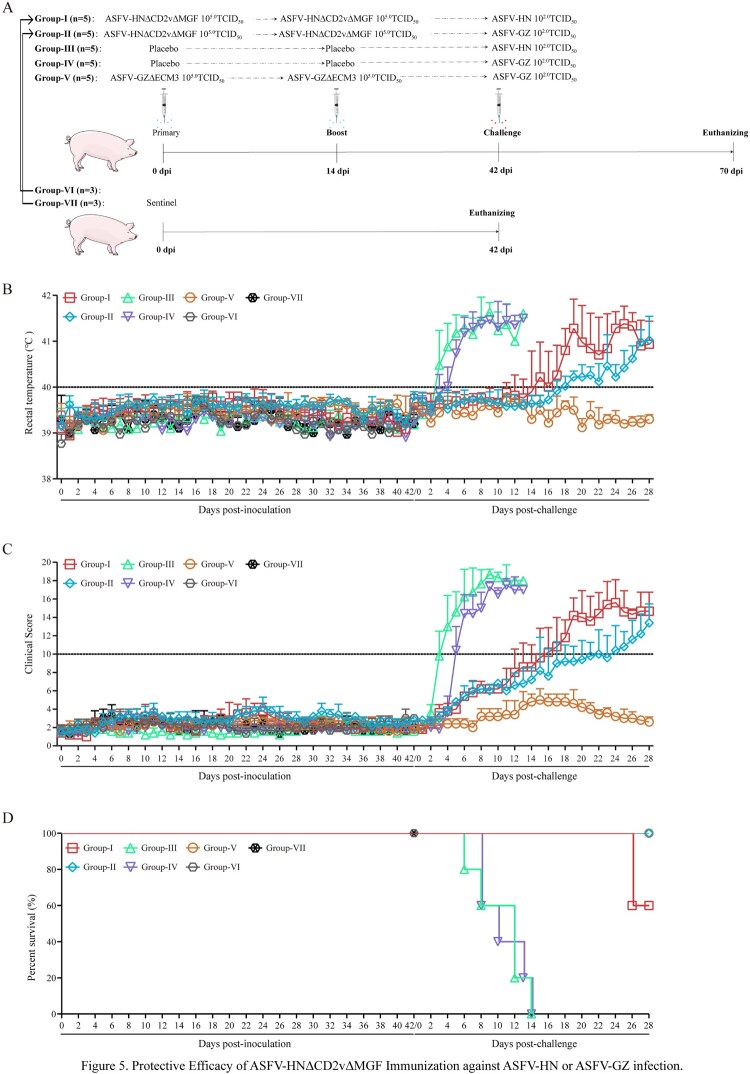
Protective Efficacy of ASFV-HNΔCD2vΔMGF Immunization against ASFV-HN or ASFV-GZ infection. (A) Timeline of animal experiments. Pigs were inoculated with placebo or 10^5.0^ TCID_50_ ASFV-HNΔCD2vΔMGF or ASFV-HNΔECM3, followed by boost immunization at 14 days post-inoculation. Lethal challenge with 10^2.0^ TCID_50_ ASFV-HN or ASFV-GZ was administered via intramuscular injection at 42 days post-inoculation. Observations continued for 28 days post-challenge prior to euthanasia. Sentinel pigs were included to monitor virus shedding in ASFV-HNΔCD2vΔMGF-immunized pigs and euthanized at the end of the observation period (42 days). (B) Rectal temperatures of pigs. (C) Clinical scores recorded and calculated of pigs. (D) Survival rates of pigs. The data are presented as means ± the standard deviations (SD). Notes: Group-I: ASFV-HNΔCD2vΔMGF-immunized, challenged with ASFV-HN; Group-II: ASFV-HNΔCD2vΔMGF-immunized, challenged with ASFV-GZ; Group-III: Placebo-immunized, challenged with ASFV-HN; Group-IV: Placebo-immunized, challenged with ASFV-GZ; Group-V: ASFV-GZΔECM3-immunized, challenged with ASFV-GZ; Group-VI: Unvaccinated, co-housed with Group-I; Group-VI: Unvaccinated, co-housed with Group-II.

To assess the protective efficacy of the ASFV-HNΔCD2vΔMGF strain in domestic pigs, the experimental groups (Group-I and Group-II) were immunized with ASFV-HNΔCD2vΔMGF; alongside them, a positive control group (Group-V) was immunized with ASFV-GZΔECM3 [22]. All immunized groups received the same two-dose regimen, and unimmunized sentinel pigs (Group-VI and Group-VII) were introduced into the study. Following two immunizations, pigs were challenged with 10^2.0^ TCID_50_ of either ASFV-HN or ASFV-GZ. A placebo (PBS)-immunized control group (Group-III and Group-IV) was concurrently established and challenged under identical conditions (Figure 5(A)). As previously described, rectal temperatures and clinical symptoms were continuously monitored. Throughout the 42-day immunization period prior to challenge, all pigs across the immunized groups (ASFV-HNΔCD2vΔMGF: Group-I and -II; ASFV-GZΔECM3: Group-V), the placebo control groups (Group-III and -IV), and the unimmunized sentinel pigs (Group-VI and -VII) remained clinically healthy, with no signs of fever or ASF-related symptoms, confirming the safety of the immunization regimen itself (Figure 5(B)–(D)). Following the challenge, however, outcomes diverged markedly between groups. All placebo-immunized pigs (Group-III and Group-IV) developed fever (>40°C) within 3–4 days post-challenge, which rapidly progressed to pronounced ASF symptoms (clinical score >10), and culminating in mortality for all animals within 14 days, confirming the virulence of the challenge strains (Figure 5(B)–(D) and Table S8). In contrast, pigs immunized with ASFV-HNΔCD2vΔMGF (Group-I and -II) exhibited a delayed but incomplete protection. Onset of fever and clinical signs was significantly postponed—to a mean of 13.8 days post-challenge in Group-I (homologous ASFV-HN challenge) and 17.6 days in Group-II (heterologous ASFV-GZ challenge). Despite this delay, all pigs in these groups eventually developed moderate to severe ASF symptoms (clinical score >10), with two pigs in Group-I requiring euthanasia due to extreme debilitation by day 26 (Figure 5(D), Table S8). Strikingly, pigs in the ASFV-GZΔECM3-positive control group (Group-V) demonstrated complete homologous protection. Throughout the entire post-challenge phase, they displayed no significant fever (<40℃) or clinical signs (clinical score <10), resulting in 100% survival and underscoring the protective efficacy achievable against the matched ASFV-GZ challenge strain.

**Figure 6.**
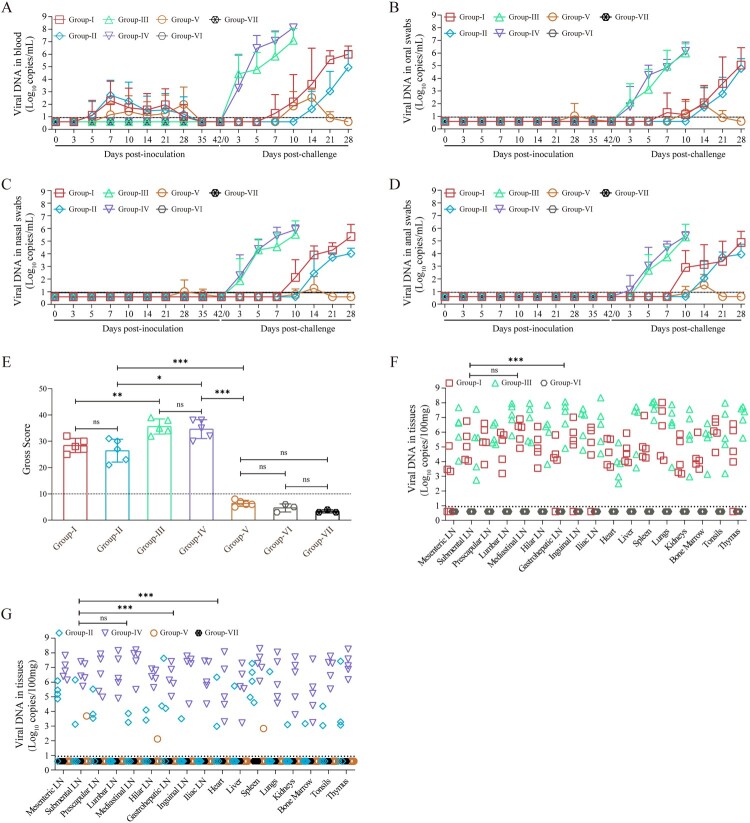
Dynamics of viral shedding and replication in pigs (A) Analysis of viremia. (B)–(D) Detection of viral shedding in oral(B), nasal(C), and anal swabs(D), respectively. (E) Pathogenic scores for all pig groups. (F and G) ASFV replication levels in tissues after challenge. LN: lymph nodes. The data are presented as means ± the standard deviations (SD). *p *<* 0.05, **p *<* *0.01*, ***p *<* *0.001,* ns: no significant. LOD = 10^0.94^ copies/mL.

As depicted in Figure 6(A), all immunized pigs, including those receiving ASFV-HNΔCD2vΔMGF (Groups I and II) or ASFV-GZΔECM3 (Group-V), developed low-level viremia during the early immunization phase. Notably, the viral genome load in the blood of all immunized animals fell below the detection limit (10^0.94^ copies/mL) between 35 and 42 days post-primary immunization. Infectious virus assays confirmed that no replicating virus could be isolated from the blood at the tested time points (14, 35, and 42 dpi)(Table S9). Analysis of oral, nasal, and anal swabs revealed no significant virus shedding (below the detection limit of 10^0.94^ copies/mL) from any of the ASFV-HNΔCD2vΔMGF-immunized pigs (Groups I and II) during the immunization period (Figure 6(B)–(D)). Importantly, no viremia was detected in co-housed sentinel pigs (Group-V and Group-VI) throughout the observation period, indicating negligible or extremely low-level virus shedding from ASFV-HNΔCD2vΔMGF-immunized pigs, insufficient to induce infection in naive animals. In the ASFV-GZΔECM3 group (Group-V), only one animal exhibited transient, low-level viral genomes in oral and nasal swabs on days 28 and 35 (Figure 6 (B) and (C) and S3 (D) and (E)).

Following challenge, the placebo-immunized control group (Group-III and Group-IV) exhibited viremia within 3–5 days of inoculation with ASFV-HN or ASFV-GZ, reaching peak viral loads (10^3.85^–10^8.24^ copies/mL) at the time of death. In ASFV-HNΔCD2vΔMGF-immunized pigs challenged with the parental ASFV-HN strain (Group-I), viremia was observed between 7 and 10 days post-challenge. In contrast, viremia appeared somewhat later (14–21 days) in pigs challenged with the virulent ASFV-GZ strain (Group-II). Nevertheless, all pigs in both Groups I and II ultimately reached blood viral loads ranging from 10^3.86^ to 10^6.90^ copies/mL by the time of euthanasia or the conclusion of the observation period (Figure 6(A) and S3(C)). This high-level viremia was associated with the consistent isolation of infectious, hemadsorbing virus from the blood at 21- and 28-days post-challenge (Table S9). In stark contrast, pigs in the ASFV-GZΔECM3-positive control group (Group-V) challenged with the homologous ASFV-GZ strain exhibited a transient viremia that peaked around day 14 post-challenge and subsequently declined to below the detection limit by the end of the observation period. Consistent with this clearance, no infectious virus was isolated from the blood at 21- or 28- days post-challenge (Figure 6(A), S3(C) and Table S9). Virus shedding was observed post-challenge in ASFV-HNΔCD2vΔMGF-immunized pigs. In Group-I, shedding was detected in most animals 7–10 days after challenge, while in Group-II, a subset of pigs began shedding at 14 days. Shedding titres increased until the endpoint. Notably, in the protected ASFV-GZΔECM3 group (Group-V), no virus shedding was detectable in any pig by the end of the observation period (Figure 6(B)–(D) and S3(D)–(F)). In conclusion, while ASFV-HNΔCD2vΔMGF immunization exhibited an acceptable safety profile, it failed to confer effective protection against lethal challenge with either the parental ASFV-HN strain or the heterologous virulent ASFV-GZ strain in domestic pigs.

### ASFV-HNΔCD2vΔMGF immunization fails to prevent organ pathology induced by parental or virulent genotype II ASFV strains

At 28 days post-challenge (70 days post-primary immunization), all pigs, including moribund animals and those exhibiting clinical signs, were euthanized and subjected to comprehensive necropsy. Organ tissue lesions were scored according to established criteria[21,23]. As depicted in Figure 6(E)–(G), placebo-immunized pigs (Group-III and Group-IV) that succumbed to ASFV-HN or ASFV-GZ challenge displayed characteristic acute ASF gross lesions, including multifocal hemorrhages, lymph node enlargement and necrosis, and widespread visceral petechiae (Gross score >10). In the ASFV-HN challenge cohort, the severity of organ damage in Group I (ASFV-HNΔCD2vΔMGF-immunized) was significantly reduced compared to that in the placebo-control Group III (mean scores: 28.4 *vs.* 35.6, *p* *<* 0.01). However, no significant difference in tissue viral load was observed between these two groups (*p* *>* 0.05). Notably, Group I still developed more severe organ lesions than the healthy sentinel pigs in Group VI *(p* *<* 0.01). A similar trend was observed in pigs challenged with ASFV-GZ. Compared to the placebo-control Group IV, Group II (ASFV-HNΔCD2vΔMGF-immunized) showed milder organ pathology (mean scores: 26.4 *vs.* 34.6, *p* *<* 0.05), yet again with no significant reduction in tissue viral load *(p* *>* 0.05). Moreover, Group II exhibited significantly more severe organ damage and higher tissue viral loads than both Group V (ASFV-GZΔECM3-immunized) and the sentinel Group VII (*p* *<* 0.01). In contrast, the gross lesion scores in Group V were statistically comparable to those of the healthy sentinel pigs in Group-VII . Collectively, these findings indicate that the genotype I/II seven-gene deletion recombinant ASFV strain ASFV-HNΔCD2vΔMGF is insufficient to suppress replication of either the homologous ASFV-HN or heterologous ASFV-GZ strain in domestic pigs and fails to prevent virus-induced organ pathology.

### Dysregulated post-challenge immune responses correlate with failed protection

**Figure 7.**
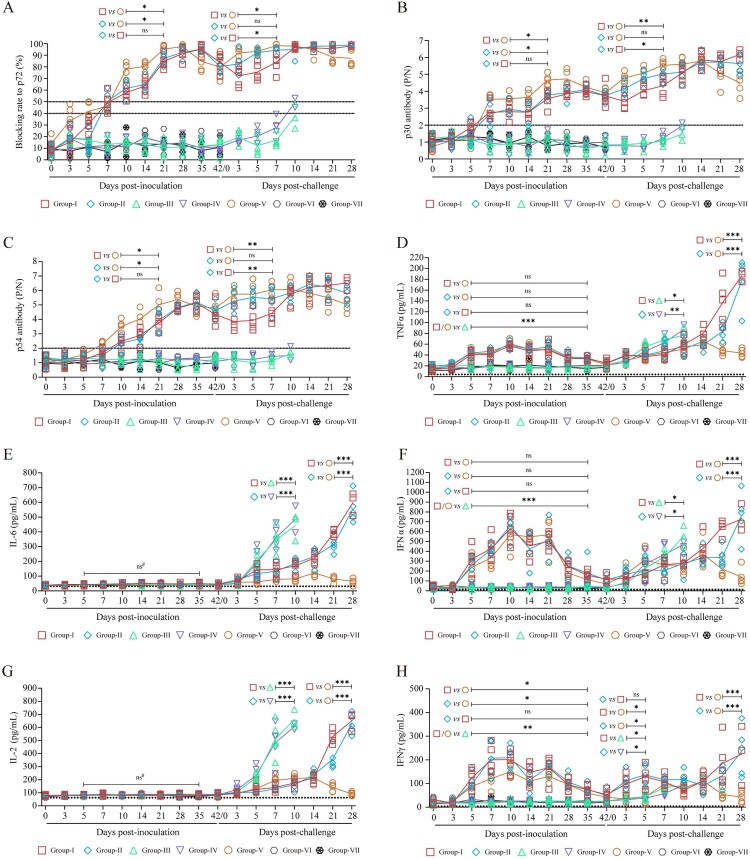
Evaluation of host immune response. (A) Detection of ASFV p72 antibody. A blocking rate of anti-p72 antibody ≥ 50% was defined as positive, ≤ 40% as negative, and between 40% and 50% was classified as suspicious. (B and C) Anti-ASFV p30 (B) or p54 (C) antibody detection. A *P/N* ratio greater than 2 is considered positive; otherwise, it is deemed negative. (D—H) Cytokine profiles in pigs at various time points: TNF-α (D, detection limit: 4 pg/mL), IL-6 (E, detection limit: 31.25 pg/mL), IFN-α (F, detection limit: 9 pg/mL), IL-2 (G, detection limit: 62.5 pg/mL), and IFN-γ (H, detection limit: 4 pg/mL). The data are presented as means ± the standard deviations (SD). *p *<* 0.05, **p *<* *0.01*, ***p *<* *0.001,* ns: no significant. ns^#^: no significant differences among all groups.

To systematically evaluate the immunogenicity and protective mechanisms of the gene-deleted ASFV strains, we analyzed the dynamics of ASFV-specific antibodies and key serum cytokines. During the pre-challenge period, ASFV-specific antibodies against p72, p30, and p54 emerged between 7- and 14- days post-inoculation (dpi) in pigs immunized with either ASFV-HNΔCD2vΔMGF (Groups I and II) or ASFV-GZΔECM3 (Group-V). Following a booster at 14 dpi, antibody levels further increased, peaking by 21–28 dpi. Notably, Group V exhibited statistically stronger antibody responses from 10 to 21 dpi compared to Groups I and II (*p* *<* 0.05), though levels had declined in all immunized animals by 42 dpi, the day of virulent challenge. After challenge, distinct antibody dynamics were observed: in Group-I (challenged with ASFV-HN), antibodies initially decreased and then increased between 0- and 7- days post-challenge (dpc), whereas Groups II and V (both challenged with ASFV-GZ) showed a consistent rise. At 3–7 dpc, Group-I exhibited significantly lower antibody levels than Groups II and V *(p* *<* 0.05), though levels converged by 10–14 dpc, after which Group V levels gradually declined. Control pigs (Groups III and IV) showed no detectable specific antibody responses post-challenge except one animal in Group-IV that seroconverted at 10 dpc, and no sentinel pigs seroconverted throughout the study (Figure 7(A)–(C)). It was notable that Group-I mounted a delayed antibody response relative to Groups II and V, and that antibody levels did not consistently correlate with protection – Group-II, despite similar post-challenge antibody levels to the protected Group-V, exhibited significantly higher viremia from 14 to 28 dpc.

Analysis of serum cytokines revealed that during the pre-challenge period (5–35 dpi), TNF-α levels in Groups I, II, and V were significantly higher than in placebo groups (*p* *<* *0.001*), with no difference between vaccinated groups. After challenge, TNF-α increased across all groups; at 7–10 dpc, placebo groups showed higher TNF-α than Groups I and II (*p* *<* *0.05*), but from 14–28 dpc, TNF-α continued rising in Groups I and II while decreasing in Group-V, leading to significantly higher concentrations in Groups I and II by 28 dpc (*p* *<* *0.001*) (Figure 7(D)). Before challenge, IL-6 and IL-2 levels were low and comparable among groups. After challenge, both cytokines increased from 3–7 dpc; at 7–10 dpc, IL-6 and IL-2 in Groups I and II were significantly lower than in controls (*p* *<* *0.001*), but from 14–28 dpc, they rose sharply in Groups I and II while steadily declining in Group-V to near-baseline by 28 dpc, showing a significant intergroup difference (*p* *<* *0.001*) (Figure 7 (E) and (G)). Interferon responses also differed: although no significant difference in IFN-α was seen pre-challenge between Group-V and Groups I/II, IFN-γ was significantly higher in Groups I and II. Post-challenge, IFN-α in placebo groups was higher than in Groups I and II at 7–10 dpc (*p* *<* 0.05), peaking before animals became moribund; from 14–28 dpc, IFN-α declined in Group-V but rose in Groups I and II, becoming significantly higher than in Group-V (*p* *<* 0.001). IFN-γ levels in Groups I and II were significantly higher than in Groups III, IV, and V as early as 3–5 dpc (*p* *<* 0.05), and their dynamics featured an initial rise, a transient decline, and a secondary rapid increase peaking before death, whereas in Group V, IFN-γ decreased gradually over 14–28 dpc, with Groups I and II becoming significantly elevated relative to Group-V (*p* *<* 0.001)(Figure 7 (F) and (H)). These findings underscore that the protective efficacy of gene-deleted ASFV vaccines depends not only on initiating immunity but, crucially, on regulating the response to prevent excessive immune-mediated damage after challenge.

## Discussion

African swine fever (ASF) poses a significant threat to the global pig industry due to the lack of a commercial vaccine [9,24–26]. The recent emergence of highly virulent genotype I/II recombinant ASFV strains in China, Vietnam, and Russia, which may become dominant, presents new challenges to conventional prevention and control measures that have traditionally focused on single-genotype viruses[4,5,7,9,27]. This study explores the potential to address this challenge by constructing two gene-deleted mutants of the genotype I/II recombinant strain ASFV-HN: ASFV-HNΔMGF (deleted for the *MGF505-1R*, *-2R*, *-3R* and *MGF360-12L*, *-13L*, *-14L* gene clusters) and ASFV-HNΔCD2vΔMGF (deleted for both the *EP402R* gene and the same *MGF360/505* gene clusters). We found that the parental ASFV-HN strain exhibited high lethality in pigs (PLD_50_ = 2.15 HAD_50_). Furthermore, while the ASFV-HNΔMGF mutant retained significant virulence, the ASFV-HNΔCD2vΔMGF mutant was significantly attenuated. All pigs inoculated with ASFV-HNΔCD2vΔMGF, whether receiving a high dose (10^5.0^ TCID_50_) or low dose (10^3.0^ TCID_50_), and regardless of single or double immunization regimens, survived and remained clinically healthy. These animals only exhibited transient, low-level viremia and no virus shedding was detected, a finding confirmed through sentinel pig co-habitation studies. Pathological examination corroborated these results: pigs infected with ASFV-HN or ASFV-HNΔMGF developed severe lesions and hemorrhagic necrosis, whereas those inoculated with ASFV-HNΔCD2vΔMGF or placebo showed no significant damage. Most critically, however, despite the excellent performance of ASFV-HNΔCD2vΔMGF immunization, it failed to provide complete protection against a lethal challenge with either the parental ASFV-HN strain or a heterologous virulent type II strain, ASFV-GZ. This indicates that while deletion of the *EP402R* and *MGF360/505* gene clusters effectively attenuates virulence, it is insufficient to elicit a protective immune response against homologous or heterologous lethal challenges.

Previous studies have indicated that deletion of the *MGF505-1R*–*MGF360-14L* cluster can attenuate the virus but may involve prolonged viremia and biosecurity risks[19,28]. In contrast, multigene-deletion strategies that include *EP402R* (CD2v) have emerged as ideal approaches. The removal of CD2v in combination with other genes significantly reduces the risk of virulence reversion[19,20] and impairs critical functions such as erythrocyte adsorption and immune evasion[29–31]. Consistent with this, a multigene-deletion backbone mutant derived from HLJ/18, with CD2v deleted, provided robust homologous protection [19]. Similarly, a recombinant strain based on ASFV-GZ with deletions in *EP402R*, *I177L*, and *MGF360* genes has also been shown to confer solid protection against homologous challenge. Our study confirms that the *EP402R* and *MGF505/360* gene clusters are key virulence determinants for the recombinant ASFV-HN strain. By constructing an attenuated strain with this combined deletion, we demonstrated its high safety in domestic pigs (immunized once or twice with 10^5.0^ or 10^3.0^ TCID_50_ doses), showing 100% survival and no clinical signs during the 28- or 42-day observation period. This conclusion aligns with Zhao et al.’s report that the JS/LG/21-7GD attenuated strain, based on a similar deletion strategy (single immunization of SPF Landrace pigs with 10^6.0^ TCID_50_), also achieved complete attenuation within a 28-day observation period [4].

Notably, the critical parallel lies in the observed lack of robust protection. Although both ASFV-HNΔCD2vΔMGF and the benchmark protective strain ASFV-GZΔECM3 successfully induced ASFV-specific antibodies against p72, p30, and p54, however, the antibody response in pigs immunized with ASFV-HNΔCD2vΔMGF was notably weaker prior to challenge. Moreover, and critically, a delayed anamnestic antibody response (3–7 dpc) was specifically observed following homologous challenge with the parental ASFV-HN strain. This early impairment in antibody re-call likely compromised initial viral control, resulting in heightened viremia and accounting for the trend toward a modestly superior protection against the heterologous ASFV-GZ strain. More importantly, antibody levels did not consistently correlate with protection, as the unprotected groups ultimately also developed high titres. The most significant difference involved immune response-related cytokines. Following virulent challenge, pigs immunized with ASFV-HNΔCD2vΔMGF exhibited a dysregulated and persistently enhanced inflammatory response. Levels of TNF-α, IL-6, IL-2, IFN-α, and IFN-γ surged in the late stages of challenge in these groups, peaking as the pigs succumbed or became moribund. Notably, the lethal “cytokine storm” occurred earlier and was more severe following homologous ASFV-HN challenge than after heterologous ASFV-GZ challenge, with the higher viremia in the former group driving this accelerated immunopathology. This pattern suggests a lethal “cytokine storm,” indicating that the vaccine-elicited immunity failed to control the challenge virus, leading to uncontrolled viral replication and consequent immunopathology[32]. In contrast, the protected group (ASFV-GZΔECM3) displayed a resolvable and controlled immune response alongside viral replication. Therefore, the reasons for protection failure are multifaceted. Additionally, ASFV encodes numerous immunosuppressive genes, and the mechanisms of immune evasion and protective immunity remain incompletely understood [8,32,33]. The significant pathogenic and immunological heterogeneity among the 24 ASFV genotypes, coupled with the emergence of recombinants, creates a complex landscape [4–6,34]. While cross-protection between some strains has been reported [35,36], its effectiveness is a complex process relying not only on sequence homology but also on the synergy between innate and adaptive immunity [33]. In summary, this failure underscores that for an ASFV vaccine, merely activating the immune system is insufficient; the quality, balance, and regulatory capacity of the elicited response are crucial to prevent lethal immunopathology upon exposure.

The failure of the ASFV-HNΔCD2vΔMGF construct to protect pigs underscores the necessity for a more cautious approach towards multigene-deleted live vaccines in the context of circulating recombinant strains. Future research directions should prioritize the following urgent tasks. First, there is a critical need to delve deeper into the core mechanisms of ASFV cross-protection to provide a scientific basis for broadly effective vaccines. This involves systematically characterizing the antigenic differences among various genotypes and recombinants and identifying the immune correlates of protection. Second, vaccine development strategies must continuously refine the selection and combination of gene targets, based on a more profound understanding of ASFV pathogenesis and immune evasion. While the current seven-gene deletion strategy represents a valuable step, it is clearly insufficient. Finally, the field must consider complementary or alternative strategies. Future efforts should integrate more rigorous safety and efficacy evaluation frameworks utilizing diverse challenge models, and explore the potential of platforms such as subunit or mRNA vaccines for achieving robust and reliable protection against the evolving spectrum of ASFV strains.

## Supplementary Material

Supplementary_material.pdf

## Data Availability

The complete genome sequences generated in this study have been deposited in GenBank under accession numbers PX667021 (parental strain ASFV-HN), PX667022 (mutant ASFV-HNΔMGF), and PX667023 (mutant ASFV-HNΔCD2vΔMGF). All data generated or analyzed during this study are included in this published article.

## References

[1] Alonso C, Borca M, Dixon L, et al. ICTV virus taxonomy profile: asfarviridae. J Gen Virol. 2018;99(5):613–614. 29565243 10.1099/jgv.0.001049 PMC12662184

[2] Zhou X, Li N, Luo Y, et al. Emergence of African swine fever in China, 2018. Transboundary Emerging Dis. 2018;65(6):1482–1484. 10.1111/tbed.12989 30102848

[3] Sun E, Huang L, Zhang X, et al. Genotype I African swine fever viruses emerged in domestic pigs in China and caused chronic infection. Emerg Microbes Infect. 2021;10(1):2183–2193. 34709128 10.1080/22221751.2021.1999779 PMC8635679

[4] Zhao D, Sun E, Huang L, et al. Highly lethal genotype I and II recombinant African swine fever viruses detected in pigs. Nat Commun. 2023;14(1):3096. 37248233 10.1038/s41467-023-38868-w PMC10226439

[5] Igolkin A, Mazloum A, Zinyakov N, et al. Detection of the first recombinant African swine fever virus (genotypes I and II) in domestic pigs in Russia. Mol Biol Rep. 2024;51(1):1011. 39320407 10.1007/s11033-024-09961-0

[6] Lee K, Vu TTH, Yeom M, et al. Molecular characterization of emerging recombinant African swine fever virus of genotype I and II in Vietnam, 2023. Emerging Microbes Infect 2024;13(1):2404156. 10.1080/22221751.2024.2404156 PMC11421136 39258419

[7] Vu HLX, McVey DS. Recent progress on gene-deleted live-attenuated African swine fever virus vaccines. npj Vaccines. 2024;9(1):60. 38480758 10.1038/s41541-024-00845-9 PMC10937926

[8] Dixon LK. Advances in African swine fever virus molecular biology and host interactions contributing to new tools for control. Favoreel HW, editor. J Virol. 2025;99(6):e00932–24. 40340396 10.1128/jvi.00932-24 PMC12172490

[9] Yang Y, Yuan H, Zhang Y, et al. Progress in African swine fever vector vaccine development. Int J Mol Sci. 2025;26(3):921. 39940691 10.3390/ijms26030921 PMC11816837

[10] Borca MV, Ramirez-Medina E, Mutisya C, et al. Evaluation of cross-protection of African swine fever vaccine ASFV-G-ΔI177L between ASFV biotypes. Vaccines. 2025;13(8):858–878. 40872943 10.3390/vaccines13080858 PMC12390177

[11] Borca MV, Rai A, Espinoza N, et al. African swine fever vaccine candidate ASFV-G-ΔI177L produced in the swine macrophage-derived cell line IPKM remains genetically stable and protective against homologous virulent challenge. Viruses. 2023;15(10):2064. 37896841 10.3390/v15102064 PMC10612016

[12] Gladue DP, Ramirez-Medina E, Vuono E, et al. Deletion of the A137R gene from the pandemic strain of African swine fever virus attenuates the strain and offers protection against the virulent pandemic virus. Shisler JL, editor. J Virol. 2021;95(21):e01139–21. 34406865 10.1128/JVI.01139-21 PMC8513468

[13] Li D, Liu Y, Qi X, et al. African swine fever virus MGF-110-9L-deficient mutant has attenuated virulence in pigs. Virol Sin. 2021;36(2):187–195. 33689140 10.1007/s12250-021-00350-6 PMC8087726

[14] Zhang J, Zhang Y, Chen T, et al. Deletion of the L7L-L11L genes attenuates ASFV and induces protection against homologous challenge. Viruses. 2021;13(2):255. 33567491 10.3390/v13020255 PMC7915138

[15] Ramirez-Medina E, Vuono E, Rai A, et al. Deletion of E184L, a putative DIVA target from the pandemic strain of african swine fever virus, produces a reduction in virulence and protection against virulent challenge. Heise MT, editor. J Virol. 2022;96(1):e01419–21. 34668772 10.1128/JVI.01419-21 PMC8754217

[16] Liu Y, Shen Z, Xie Z, et al. African swine fever virus I73R is a critical virulence-related gene: a potential target for attenuation. Proc Natl Acad Sci. 2023;120(15):e2210808120. 37023125 10.1073/pnas.2210808120 PMC10104517

[17] Huang R, Luo R, Lan J, et al. The multigene family genes-encoded proteins of African swine fever virus: roles in evolution, cell tropism, immune evasion, and pathogenesis. Viruses. 2025;17(6):865. 40573456 10.3390/v17060865 PMC12197721

[18] Borca MV, O’Donnell V, Holinka LG, et al. Deletion of CD2-like gene from the genome of African swine fever virus strain Georgia does not attenuate virulence in swine. Sci Rep. 2020;10(1):494. 31949276 10.1038/s41598-020-57455-3 PMC6965178

[19] Rathakrishnan A, Connell S, Petrovan V, et al. Differential effect of deleting members of African swine fever virus multigene families 360 and 505 from the genotype II Georgia 2007/1 isolate on virus replication, virulence, and induction of protection. Heise MT, editor. J Virol. 2022;96(6):e01899–21. 35044212 10.1128/jvi.01899-21 PMC8941908

[20] Chen W, Zhao D, He X, et al. A seven-gene-deleted African swine fever virus is safe and effective as a live attenuated vaccine in pigs. Sci China Life Sci. 2020;63(5):623–634. 32124180 10.1007/s11427-020-1657-9 PMC7223596

[21] Wang Z, Zhang J, Li F, et al. The attenuated African swine fever vaccine HLJ/18-7GD provides protection against emerging prevalent genotype II variants in China. Emerg Microbes Infect. 2024;13(1):2300464. 38164797 10.1080/22221751.2023.2300464 PMC10810661

[22] Liu Y, Li Y, Xie Z, et al. Development and in vivo evaluation of MGF100-1R deletion mutant in an African swine fever virus Chinese strain. Vet Microbiol. 2021;261:109208. 34419775 10.1016/j.vetmic.2021.109208

[23] Xie Z, Liu Y, Di D, et al. Protection evaluation of a five-gene-deleted African swine fever virus vaccine candidate against homologous challenge. Front Microbiol. 2022;13:902932. 35966648 10.3389/fmicb.2022.902932 PMC9374035

[24] Liu Y, Xie Z, Li Y, et al. Evaluation of an I177L gene-based five-gene-deleted African swine fever virus as a live attenuated vaccine in pigs. Emerg Microbes Infect. 2023;12(1):2148560. 36378022 10.1080/22221751.2022.2148560 PMC9769145

[25] Blome S, Gabriel C, Beer M. Modern adjuvants do not enhance the efficacy of an inactivated African swine fever virus vaccine preparation. Vaccine. 2014;32(31):3879–3882. 24877766 10.1016/j.vaccine.2014.05.051

[26] Teklue T, Sun Y, Abid M, et al. Current status and evolving approaches to African swine fever vaccine development. Transbound Emerg Dis. 2020;67(2):529–542. 31538406 10.1111/tbed.13364

[27] Zhang H, Zhao S, Zhang H, et al. Vaccines for African swine fever: an update. Front Microbiol. 2023;14:1139494. 37180260 10.3389/fmicb.2023.1139494 PMC10173882

[28] Le VP, Nguyen VT, Le TB, et al. Detection of recombinant African swine fever virus strains of p72 genotypes I and II in domestic pigs, Vietnam, 2023. Emerg Infect Dis. 2024;30(5):991–994. 38666642 10.3201/eid3005.231775 PMC11060461

[29] O’Donnell V, Holinka LG, Gladue DP, et al. African swine fever virus Georgia isolate harboring deletions of MGF360 and MGF505 genes is attenuated in swine and confers protection against challenge with virulent parental virus. Perlman S, editor. J Virol. 2015;89(11):6048–6056. 25810553 10.1128/JVI.00554-15 PMC4442422

[30] Rodríguez JM, Yáñez RJ, Almazán F, et al. African swine fever virus encodes a CD2 homolog responsible for the adhesion of erythrocytes to infected cells. J Virol. 1993;67(9):5312–5320. 8102411 10.1128/jvi.67.9.5312-5320.1993 PMC237930

[31] Gao Q, Yang Y, Luo Y, et al. African swine fever virus envelope glycoprotein CD2v interacts with host CSF2RA to regulate the JAK2-STAT3 pathway and inhibit apoptosis to facilitate virus replication. Jung JU, editor. J Virol. 2023;97(4):e01889–22. 37022174 10.1128/jvi.01889-22 PMC10134862

[32] Borca MV, Ramirez-Medina E, Espinoza N, et al. Deletion of the EP402R gene from the genome of African swine fever vaccine strain ASFV-G-ΔI177L provides the potential capability of differentiating between infected and vaccinated animals. Viruses. 2024;16(3):376. 38543742 10.3390/v16030376 PMC10974803

[33] Solikhah TI, Rostiani F, Nanra AFP, et al. African swine fever virus: virology, pathogenesis, clinical impact, and global control strategies. Vet World. 2025;18(6):1599–1613. 40689197 10.14202/vetworld.2025.1599-1613 PMC12269935

[34] Zhang T, Lu Z, Liu J, et al. Host innate and adaptive immunity against African swine fever virus infection. Vaccines. 2024;12(11):1278. 39591181 10.3390/vaccines12111278 PMC11599025

[35] Qu H, Ge S, Zhang Y, et al. A systematic review of genotypes and serogroups of African swine fever virus. Virus Genes. 2022;58(2):77–87. 35061204 10.1007/s11262-021-01879-0 PMC8778497

[36] Monteagudo PL, Lacasta A, López E, et al. BA71 CD2: a new recombinant live attenuated African swine fever virus with cross-protective capabilities. J Virol. 2017;91(21):e01058–17. 28814514 10.1128/JVI.01058-17 PMC5640839

