# Supplementary material for: The deletion of the EP402R and MGF505/360 genes attenuates a genotype I/II recombinant ASFV but fails to confer complete protection against homologous or genotype II challenge in pigs
Source: Emerg Microbes Infect. 2026 Jan 12;15(1):2608396. doi: 10.1080/22221751.2025.2608396 (PMC12798666; doi:10.1080/22221751.2025.2608396)
Supplement: Supplementary_material.pdf [file TEMI_A_2608396_SM4266.pdf]

Table S1. Homology of each fragment of recombinant ASFV-HN with the corresponding fragment of JS/LG/21.

**Fragment**				**Likely parent**	**Identity (%) with that of JS/LG/21(Genotype I/II)**
**ID**	**Position**		**Length (bp)**		
	**Start**	**End**			
F1	1	19,273	19,273	Genotype I	99.995%
F2	19,274	33,248	13,975	Genotype II	99.993%
F3	33,249	59,378	26,130	Genotype I	99.985%
F4	59,379	63,494	4116	Genotype II	100%
F5	63,495	66,708	3214	Genotype I	100%
F6	66,709	77,562	10,854	Genotype II	99.991%
F7	77,563	81,254	3692	Genotype I	100%
F8	81,255	86,883	5629	Genotype II	100%
F9	86,884	102,453	15,570	Genotype I	99.981%
F10	102,454	115,776	13,323	Genotype II	99.992%
F11	115,777	118,739	2963	Genotype I	100%
F12	118,740	128,884	10,145	Genotype II	100%
F13	128,885	131,665	2781	Genotype I	100%
F14	131,666	136,729	5064	Genotype II	100%
F15	136,730	137,277	548	Genotype I	100%
F16	137,278	140,785	3508	Genotype II	100%
F17	140,786	142,184	1399	Genotype I	100%
F18	142,185	149,814	7630	Genotype II	100%
F19	149,815	154,854	5040	Genotype I	100%
F20	154,855	185,431	30,577	Genotype II	99.974%

A

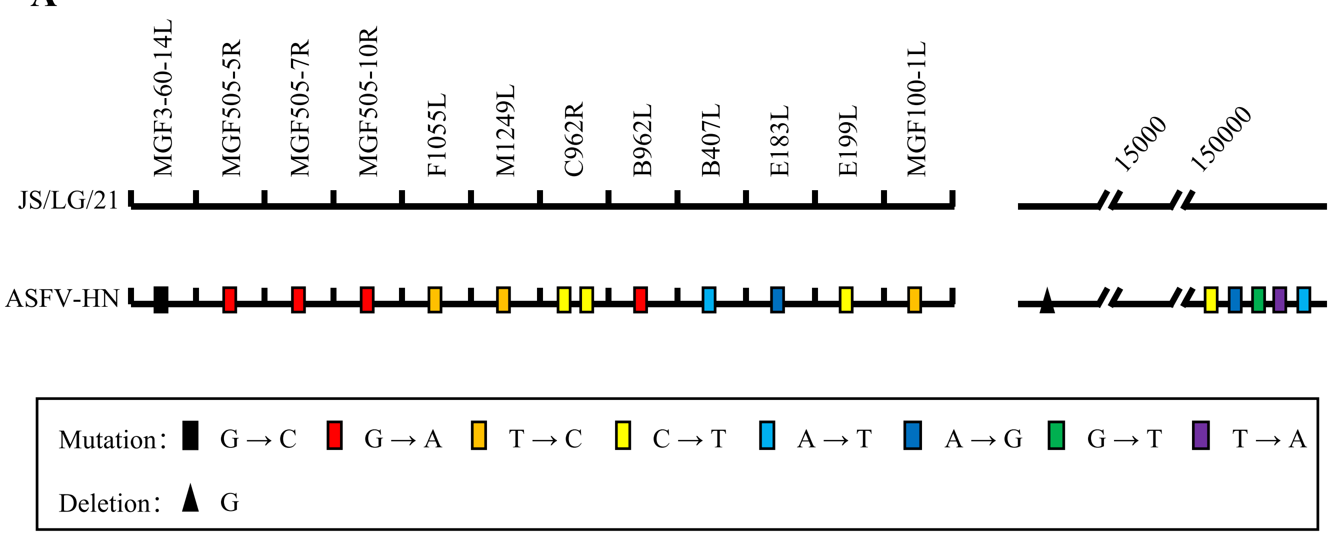

Figure S1. Genetic changes in the recombinant ASFV-HN compared with the genotype I/II recombinant JS/LG/21.

Table S2. Genetic changes in the recombinant ASFV-HN compared with the genotype I/II recombinant JS/LG/21.

Virus	Position	ORF/Region	Nucleotide change	ORF/Amino acid change
	15744	Noncoding Region	G deletion	/
	28226	MGF360-14L	G → C	A → G
	33351	MGF505-5R	G → A	/
	35982	MGF505-7R	G → A	S→ N
	40524	MGF505-10R	G → A	D→ N
	57723	F1055L	T → C	K→ E
	71256	M1249L	T → C	D→ G
	87430	C962R	C → T	A→ V
	87433	C962R	C → T	P→ L
ASFV-HN	90071	B962L	G → A	/
	102856	B407L	A → T	/
	158646	E183L	A → G	/
	162007	E199L	C → T	E→ K
	168280	Noncoding Region	C → T	/
	175540	MGF100-1L	T → C	Y→ C
	185318	Noncoding Region	A → G	/
	185347	Noncoding Region	G → T	/
	185374	Noncoding Region	T → A	/
	185378	Noncoding Region	A → T	/

/ means no change.

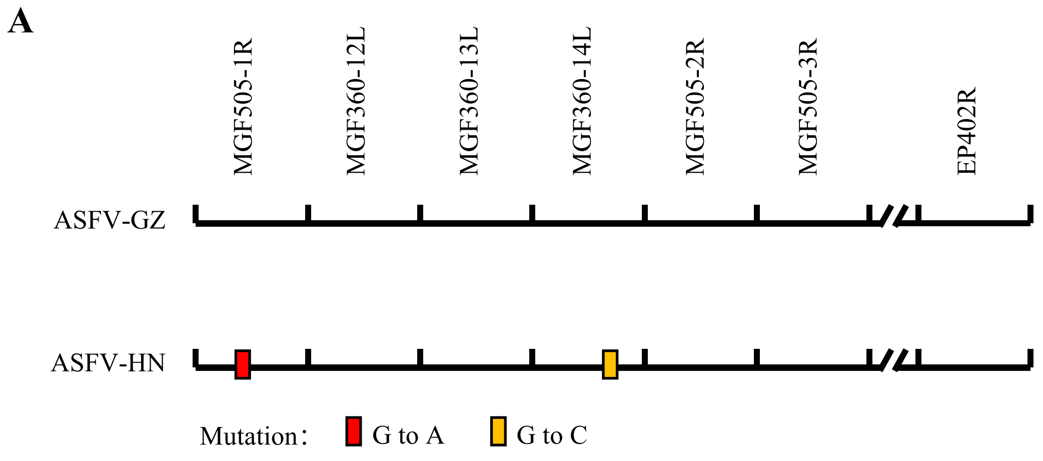

Figure S2. *MGF505/360* and *EP402R* changes in the recombinant ASFV-HN compared with genotype II ASFV-GZ.

Table S3. *MGF505/360* and *EP402R* in the ASFV-HN compared with ASFV-GZ.

Virus	Position	ORF/Region	Nucleotide change	ORF/Amino acid change
	23545	MGF505-1R	G → A	R → K
	24892	MGF360-12L	/	/
	26105	MGF360-13L	/	/
ASFV-HN	28226	MGF360-14L	G → C	A → G
	28631	MGF505-2R	/	/
	30298	MGF505-3R	/	/
	68879	EP402R	/	/

/ means no change.

Table S4. Genetic changes in the recombinant ASFV-HNΔMGF compared with the ASFV-HN.

Virus	Position	ORF/Region	Nucleotide change	ORF/Amino acid change
	5569	Noncoding Region	T → A	/
	6619	Noncoding Region	A → T	/
	9865	MGF100-1R	T → A	F → Y
	23244	MGF505-1R	gene deletion	complete deletion
	24892	MGF360-12L	gene deletion	complete deletion
	26105	MGF360-13L	gene deletion	complete deletion
ASFV-HN ΔMGF	27562	MGF360-14L	gene deletion	complete deletion
	28631	MGF505-2R	gene deletion	complete deletion
	30298	MGF505-3R	gene deletion	complete deletion
	32310	MGF505-10R	A → T	K → M
	171422	MGF360-18R	A → G	E → G
	174631	Noncoding Region	C → G	/
	184502	Noncoding Region	A deletion	/

/ means no change.

Table S5. Genetic changes in the recombinant ASFV-HNΔCD2vΔMGF compared with the ASFV-HN.

Virus	Position	ORF/Region	Nucleotide change	ORF/Amino acid change
ASFVHN ΔCD2vΔMGF	2968	Noncoding Region	C → T	/
	18245	MGF300-4L	A → G	/
	20845	Noncoding Region	T → G	/
	20863	Noncoding Region	A → T	/
	22856	MGF360-11L	T → A	L → F
	23244	MGF505-1R	gene deletion	complete deletion
	24892	MGF360-12L	gene deletion	complete deletion
	26105	MGF360-13L	gene deletion	complete deletion
	27562	MGF360-14L	gene deletion	complete deletion
	28631	MGF505-2R	gene deletion	complete deletion
	30298	MGF505-3R	gene deletion	complete deletion
	68879	CD2v(EP402R)	gene deletion	complete deletion
	159455	Noncoding Region	A → T	/
	160627	Noncoding Region	G → T	/
	164190	MGF360-16R	C → T	H → Y
	166802	MGF100-1L	A → G	/
	170701	MGF360-18R	G → C	A → P

/ means no change.

Table S6. Swine survival and clinical presentations after inoculated with with ASFV-HN, ASFV-HNΔMGF or ASFV-HNΔCD2vΔMGF.

Groups	No. of survivors/total	Time to death [Mean(SD)] (days)	Data for fever [mean (SD)]		
			onset (days)	Duration (days)	Maximum rectal temp (°C)
$10^{5.0}$TCID$_{50}$ ASFV-HN	0/5	5.6(1.14)	2.8(0.45)	3.14(1.14)	42.0
$10^{3.0}$TCID$_{50}$ ASFV-HN	0/5	7.8(0.84)	3.8(0.45)	4.8(1.30)	41.8
$10^{5.0}$TCID$_{50}$ ASFV-HNΔMGF	0/5	14.8(3.11)	4.2(0.45)	3.4(1.14)	41.8
$10^{3.0}$TCID$_{50}$ ASFV-HNΔMGF	3/5	21(5.66)	4.8(0.84)	14.2(4.76)	41.6
$10^{5.0}$TCID$_{50}$ ASFV-HNΔCD2vΔMGF	5/5	/	/	/	39.9
$10^{3.0}$TCID$_{50}$ ASFV-HNΔCD2vΔMGF	5/5	/	/	/	39.9

Table S7. The pathogenic scores of the infected tissues from the pigs inoculated with ASFV-HN, ASFV-HNΔMGF or ASFV-HNΔCD2vΔMGF or the placebo control group.

Groups	Pig NO.	Gross lesions of the tissues observed during necropsy.Severity &Scoring:Mild(1),Moderate(2),Severe(3)								
		Tonsil	Kidney	Liver	Spleen	Lung	Thymus	LNs†	Gastrointestine	Joint
$10^{5.0}TCID_{50}$ ASFV-HN	HN-1	Extravasated blood(2)	Hemorrhage (3)	Hyperemia(1)	Petechia(3)	Extravasated blood(2)	Petechia(2)	Hyperemia, Swell(24)	Cecum Hemorrhage (1)	Effusion (1)
	HN-2	*	Hemorrhage(1)	Hyperemia(1)	Petechia(3)	Extravasated blood(1)	Petechia(1)	Hyperemia, Swell(25)	Cecum Hemorrhage (1)	*
	HN-3	Extravasated blood(3)	Hemorrhage(2)	Hyperemia(1)	Hyperemia(2)	Edema(1)	Petechia(1)	Hyperemia, Swell(24)	Gastric mucosal hemorrhage(2)	Swell(1)
	HN-4	*	Hemorrhage(3)	*	Petechia(2)	Edema(2)	Petechia(2)	Hyperemia, Swell(20)	*	Effusion (1)
	HN-5	Extravasated blood(2)	Hemorrhage(2)	*	Hyperemia(3)	Extravasated blood(1)	Hyperemia(3)	Hyperemia, Swell(26)	*	*
$10^{3.0}TCID_{50}$ ASFV-HN	HN-6	*	*	Hyperemia(1)	Petechia(3)	*	*	Hyperemia, Swell(24)	*	*
	HN-7	Extravasated blood(1)	Hemorrhage(2)	Hyperemia(1)	Petechia(3)	Edema(1)	*	Hyperemia, Swell(25)	*	Swell(1)
	HN-8	Flush(1)	Hemorrhage(2)	*	Petechia(2)	Edema(1)	Hyperemia(3)	Hyperemia, Swell(24)	*	Swell(2)
	HN-9	Extravasated blood(2)	*	Hyperemia(1)	Petechia(3)	Extravasated blood(1)	Hyperemia(3)	Hyperemia, Swell(26)	Cecum Hemorrhage (1)	*
	HN-10	Extravasated blood(3)	Hemorhage(3)	*	Petechia(3)	Edema(2)	Petechia(1)	Hyperemia, Swell(26)	*	Swell(1)
$10^{5.0}TCID_{50}$ ASFV-HNΔMGF	A1	Extravasated blood(3)	*	Hyperemia(1)	Hyperemia(3)	Extravasated blood(2)	Petechia(3)	Hyperemia, Swell(24)	*	Swell(1)
	A2	Extravasated blood(1)	Hemorrhage(2)	Hyperemia(1)	Hyperemia(3)	Extravasated blood(2)	Hyperemia(1)	Hyperemia, Swell(24)	Cecum Hemorrhage (1)	*
	A3	Flush(1)	Hemorrhage(2)	*	Petechia(3)	Extravasated blood(2)	*	Hyperemia, Swell(19)	Cecum Hemorrhage (2)	Effusion (1)
	A4	*	Hemorrhage(2)	*	Petechia(3)	*	*	Hyperemia, Swell(19)	*	*
	A5	Flush(1)	*	*	Petechia(3)	Edema(2)	Petechia(3)	Hyperemia, Swell(24)	*	Swell(1)
$10^{3.0}TCID_{50}$ ASFV-HNΔMGF	A6	*	*	*	Hyperemia(2)	*	Petechia(2)	Hyperemia, Swell(18)	*	*
	A7	Flush(1)	Hemorrhage(2)	*	Hyperemia(2)	*	Petechia(2)	Hyperemia, Swell(15)	Cecum Hemorrhage (1)	*
	A8	Extravasated blood(3)	Hemorrhage(2)	Hyperemia(1)	Petechia(3)	*	Petechia(2)	Hyperemia, Swell(22)	*	*
	A9	Extravasated blood(3)	Hemorrhage(2)	Hyperemia(1)	Petechia(3)	Edema(1)	Hyperemia(3)	Hyperemia, Swell(23)	*	*
	A10	*	*	*	Hyperemia(2)	Edema(1)	*	Hyperemia, Swell(17)	*	*
$10^{5.0}TCID_{50}$ ASFV-HNΔCD2vΔMGF	B1	*	*	*	*	*	*	Hyperemia, Swell(9)	*	*
	B2	*	*	*	*	*	*	Hyperemia, Swell(6)	*	*
	B3	*	*	*	*	*	*	Hyperemia, Swell(4)	*	*
	B4	*	*	*	*	*	*	Hyperemia, Swell(8)	*	*
	B5	*	*	*	*	*	*	Hyperemia, Swell(4)	*	*
$10^{3.0}TCID_{50}$ ASFV-HNΔCD2vΔMGF	B6	*	*	*	*	*	*	Hyperemia, Swell(2)	*	*
	B7	*	*	*	*	*	*	Hyperemia, Swell(4)	*	*
	B8	*	*	*	*	*	*	Hyperemia, Swell(2)	*	*
	B9	*	*	*	*	*	*	Hyperemia, Swell(3)	*	*
	B10	*	*	*	*	*	*	Hyperemia, Swell(3)	*	*
Placebo	N1	*	*	*	*	*	*	Hyperemia, Swell(3)	*	*
	N2	*	*	*	*	*	*	Hyperemia, Swell(2)	*	*
	N3	*	*	*	*	*	*	Hyperemia, Swell(2)	*	*

*No gross lesion observed.
†Nine kinds of LNs were included: mesenteric LNs, submental LNs, prescapular LNs, lumbar LNs, mediastinal LNs, hilar LNs, gastrohepatic LNs, inguinal LNs, and iliac LN.

Table S8. Swine survival and clinical presentations after challenge in efficacy experiments.

Groups	Description	No. of survivors/total	Time to death [Mean (SD)] (days)	Data for fever [mean (SD)]		
				onset (days)	Duration (days)	Maximum rectal temp (°C)
I	Immunized twice with $10^{5.0}$ $TCID_{50}$ of ASFV-HNΔCD2vΔMGF and challenged with $10^{2.0}$ $TCID_{50}$ of ASFV-HN.	3/5*	26(0)	13.8(3.42)	7.8(2.39)	41.9
II	Immunized twice with $10^{5.0}$ $TCID_{50}$ of ASFV-HNΔCD2vΔMGF and challenged with $10^{2.0}$ $TCID_{50}$ of ASFV-GZ.	5/5*	/	17.6(0.89)	6.2(1.10)	41.8
III	Immunized twice with placebo (PBS) and challenged with $10^{2.0}$ $TCID_{50}$ of ASFV-HN.	0/5	10.4(3.29)	3(0.71)	7.4(3.78)	41.8
IV	Immunized twice with placebo (PBS) and challenged with $10^{2.0}$ $TCID_{50}$ of ASFV-GZ.	0/5	10.6(2.79)	4.4(0.89)	6.2(3.11)	41.7
V	Immunized twice with $10^{5.0}$ $TCID_{50}$ of ASFV-GZΔECM3 and challenged with $10^{2.0}$ $TCID_{50}$ of ASFV-GZ.	5/5#	/	/	/	39.9
VI	Sentinel pigs: Unvaccinated, co-housed with Group-I.	3/3#	/	/	/	39.8
VII	Sentinel pigs: Unvaccinated, co-housed with Group-II.	3/3#	/	/	/	39.7

*Survived but showed clinical ASF signs.
#Survived without clinical ASF signs.

Table S9. Analysis of Infectious Virus in Blood Samples from the Vaccinated Group.

Groups	Description	Pig NO.	Days pre-challenge			Days post-challenge	
			14	35	42	21	28
I	Immunized twice with $10^{5.0}$ $TCID_{50}$ of ASFV-HNΔCD2vΔMGF and challenged with $10^{2.0}$ $TCID_{50}$ of ASFV-HN.	A1	-	-	-	+	/
		A2	-	-	-	+	+
		A3	-	-	-	+	+
		A4	-	-	-	+	/
		A5	-	-	-	+	+
II	Immunized twice with $10^{5.0}$ $TCID_{50}$ of ASFV-HNΔCD2vΔMGF and challenged with $10^{2.0}$ $TCID_{50}$ of ASFV-GZ.	B1	-	-	-	+	+
		B2	-	-	-	-	+
		B3	-	-	-	+	+
		B4	-	-	-	-	+
		B5	-	-	-	+	+
V	Immunized twice with $10^{5.0}$ $TCID_{50}$ of ASFV-GZΔECM3 and challenged with $10^{2.0}$ $TCID_{50}$ of ASFV-GZ.	E1	-	-	-	-	-
		E2	-	-	-	-	-
		E3	-	-	-	-	-
		E4	-	-	-	-	-
		E5	-	-	-	-	-

- no virus isolated; + virus isolated; / deceased animal.
